# The model student: GPT-4 performance on graduate biomedical science exams

**DOI:** 10.1038/s41598-024-55568-7

**Published:** 2024-03-07

**Authors:** Daniel Stribling, Yuxing Xia, Maha K. Amer, Kiley S. Graim, Connie J. Mulligan, Rolf Renne

**Affiliations:** 1 Department of Molecular Genetics and Microbiology, University of Florida, Gainesville, FL 32610 USA; 2 UF Genetics Institute, University of Florida, Gainesville, FL 32610 USA; 3 UF Health Cancer Center, University of Florida, Gainesville, FL 32610 USA; 4 Department of Neuroscience, Center for Translational Research in Neurodegenerative Disease, College of Medicine, University of Florida, Gainesville, FL 32610 USA; 5 Department of Computer and Information Science and Engineering, Herbert Wertheim College of Engineering, University of Florida, Gainesville, FL 32610 USA; 6 Department of Anthropology, University of Florida, Gainesville, FL 32610 USA; 7 Present Address: Department of Neurology, UCLA, Los Angeles, CA 90095 USA

**Keywords:** Computer science, Software

## Abstract

The GPT-4 large language model (LLM) and ChatGPT chatbot have emerged as accessible and capable tools for generating English-language text in a variety of formats. GPT-4 has previously performed well when applied to questions from multiple standardized examinations. However, further evaluation of trustworthiness and accuracy of GPT-4 responses across various knowledge domains is essential before its use as a reference resource. Here, we assess GPT-4 performance on nine graduate-level examinations in the biomedical sciences (seven blinded), finding that GPT-4 scores exceed the student average in seven of nine cases and exceed all student scores for four exams. GPT-4 performed very well on fill-in-the-blank, short-answer, and essay questions, and correctly answered several questions on figures sourced from published manuscripts. Conversely, GPT-4 performed poorly on questions with figures containing simulated data and those requiring a hand-drawn answer. Two GPT-4 answer-sets were flagged as plagiarism based on answer similarity and some model responses included detailed hallucinations. In addition to assessing GPT-4 performance, we discuss patterns and limitations in GPT-4 capabilities with the goal of informing design of future academic examinations in the chatbot era.

## Introduction

Large language model (LLM) artificial intelligence (AI) methods have emerged as an impactful and disruptive influence on many language-based tasks that have traditionally been the sole domain of humans^1–3^. In November 2022, OpenAI released the Chat Generative Pre-Trained Transformer (ChatGPT) chatbot to the general public via a web interface, providing wide accessibility to the GPT-3.5 LLM and drastically reducing the barriers to its use^4,5^. GPT-3.5 improved upon its predecessors by incorporating “reinforcement learning with human feedback” concepts from the OpenAI InstructGPT sister model^5,6^, which allowed it to provide responses more aligned with the intentions of its queries than its predecessors^5^. ChatGPT marked a notable increase in the apparent capability of LLMs and has been considered by many as a significant improvement over other available models. Since its release, ChatGPT and other LLMs (Google Bard, Perplexity) have driven broad adoption of text-generation technology for tasks across a variety of fields and domains^7–9^. One notable example is university students’ rapid adoption of ChatGPT to complete essays and assignments, disrupting many existing assessment paradigms in higher education^7,10,11^.

One of the major improvements of GPT-3.5 over its predecessors is its ability to answer a higher proportion of questions with factually correct responses containing logically consistent reasoning. The GPT-4 model released by OpenAI in March 2023 (incorporated as an option for ChatGPT shortly after) further builds on this capability with improvements in the generation of answers containing stepwise logical and critical thinking^3,12^. Given the GPT models’ design as general-purpose tools for creating human-like text, it is enticing to consider that successive iterations of ChatGPT may function as capable “answer engines” for accessing and formatting factual information. However, one of the known limitations of the GPT models (and LLMs in general) is their propensity to “hallucinate,” where the model provides responses with fictional or incorrect information with the same confidence as factually correct answers^5,12–15^. This characteristic stems from the nature of these models’ design and training: their primary functionality of learning patterns of words in English language text and the stochastic nature in which LLM responses are generated^3^. One prominent example of hallucinations by ChatGPT is the generation of realistic citations to fictional reference sources^3,13,16,17^, which has already resulted in GPT-generated references to nonexistent legal cases being submitted in a legal brief in New York^18^ (though ChatGPT now accompanies some fictional citations with a disclaimer as shown in Supplementary Fig. 1).

As ChatGPT and GPT-4 have demonstrated significant potential and are already being adopted in disciplines requiring accurate outputs, it is critical to characterize response quality across multiple knowledge domains^18–20^. To this end, LLM performance on multiple choice questions from both broad-based and discipline-specific standardized examinations have been used as benchmarks of model knowledgebase and capabilities. These include the bar exam for law and the United States Medical Licensing Examinations (USMLE) in medicine^19,21,22^. For these exams, the GPT-3.5 model received a failing performance on the bar exam^23^ and scored at or near the passing threshold of the medical licensing exams^19,21^. In contrast, GPT-4 received a passing score on the Bar (at the 90th percentile)^12^ and scored approximately 20 percentage points over the pass threshold for the USMLE medical examination questions^22^, a remarkable improvement given the short time between release of these models. Other trainee assessments have also been used, including questions in human genetics^24^, medical training curricula^25,26^, selected examination questions^27^, and questions from professional certifications and board examinations in several medical fields^28–42^. However, standardized examinations often have extensive study resources available online for trainees, including large sets of example questions and answers. As these study materials may have been incorporated into the GPT-4 training data such as the Common Crawl^43^, standardized examinations may not be an accurate assessment of domain-specific model knowledgebase and capability. Further, if evaluation datasets depend heavily upon “sample” questions for a given assessment, the question set (and thus results) may not reflect the depth and distribution of topics within an actual instance of the respective exam^44^. Therefore, further benchmarks should be used to assess GPT-4 performance outside of the standardized examination context.

In this study, we assess the capability of GPT-4 to answer questions from nine graduate-level final examinations for scientific trainees in the biomedical sciences. In this field, academic courses required for training toward a Doctor of Philosophy (Ph.D.) degree typically involve free-response questions requiring both background knowledge and critical thinking skills. Therefore, we expect these examinations to provide a strong benchmark of GPT-4’s ability to provide correct and logically consistent responses to expert-level questions. We examine the impact of multiple styles of GPT-4 query (prompt patterns) and directly compare results to student performance. To reduce potential bias in GPT-4 answer evaluation, grading is performed blinded for most examinations. In most cases, we find that one or all sets of GPT-4 answers meet or exceed the average score of students in the course, with all GPT-4 scores exceeding all student grades for several courses. We also describe examples where GPT-4 answers compare poorly to student grades and instances in which similar answers are flagged for plagiarism. These results provide a further metric for the capability and accuracy of GPT-4 answers in scientific contexts, focusing on a broad array of biomedical disciplines using question types outside of standardized exam materials. Additionally, our evaluation of GPT-4’s capability to answer graduate-level examination questions helps inform the design of future examinations in the chatbot era and mitigate potential student misuse of LLMs.

## Results

### Participating courses

**Figure 1:**
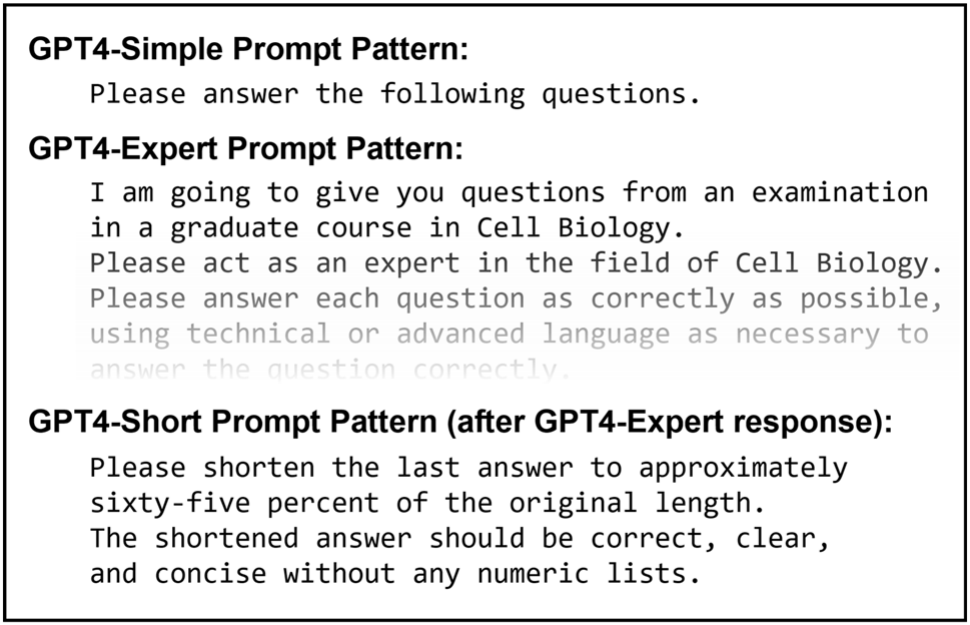
Prompt patterns used for GPT-4 queries. Example prompt patterns used to initiate a query to GPT-4 before providing examination questions. GPT4-Simple Prompt Pattern: Follows a zero-shot approach without providing any specific instructions as to answer format; GPT4-Expert Prompt Pattern: Follows a persona prompt pattern, instructing GPT-4 to act as an expert in the respective content domain and providing detailed instructions as to answer formatting; GPT4-Short: Modifies the GPT4-Expert approach by instructing that each answer be shortened to approximately 65% of initial length.

Nine courses were recruited to participate in this study, covering the domains of virology, microbiology, cellular physiology, genetics and genomics, bioinformatics, molecular biology, cancer epidemiology, biostatistics, and genetic ethics. GPT-4 answers to exam questions were obtained using 1–3 different approaches in parallel as shown in Table 1. For the “GPT4-Simple” approach, we used a minimal prompt before providing exam questions to GPT4, whereas for the GPT4-Expert and GPT4-Short approaches we used a persona prompt pattern with instructions to act as an “expert” in the relevant field and included extensive instructions as to answer length and formatting as shown in Fig. 1 (see “Methods” section for details and Supplementary Methods for full examples of prompt patterns used). Exam questions were primarily short response (1/4 to 1/2 page), with additional types including fill-in-the-blank, essay, and diagram-drawing questions. Some questions included a graphical scientific figure or diagram as key information. As multimodal input to GPT models was not yet publicly available at the time of the study, each figure or diagram was converted to a textual description for question administration (described in “Methods” section)^45^. For each course, one to three sets of GPT-4 answers were evaluated by course instructors in the same format as used by course graduate students (N = 2–8), with blinded grading performed in parallel with student exams in 7 of 9 examinations. Following grading (and any unblinding), professors provided anonymous student performance information for comparison to GPT-4 scores and answered a survey providing opinions on GPT-4 and its potential future impacts on academic courses.

**Table 1:** Graduate courses with examinations completed by GPT-4.

Course	Exam type	Blinding	Access	Figures	Format	Simple	Expert	Short
GMS6035 Advanced Virology II: RNA Viruses	Final	Partial	ChatGPT	**X**	Paper		**X**	
GMS6038: Bacterial Genetics and Physiology	Final	None	GPT-4 API		Text	**X**	**X**	**X**
GMS6473: Fund. of Phys. & Fun. Genomics	Final	Full	GPT-4 API		Text	**X**	**X**	**X**
PHC7007: Cancer Epidemiology	Final	Full	GPT-4 API		Text	**X**	**X**	**X**
GMS6231: Genomics and Bioinformatics	First-year	Full	GPT-4 API		Text	**X**	**X**	
PCB5065: Advanced Genetics	First-year	Full	GPT-4 API	**X**	Text	**X**	**X**	
BCH6415: Adv. Molecular and Cell Biology	First-year	Full	GPT-4 API		Text	**X**	**X**	
GMS6221: Genetical Ethics	First-year	Full	GPT-4 API		Text	**X**	**X**	
PHC6052: Intro. to Biostatistical Methods	First-year	Full	GPT-4 API		Text	**X**	**X**	

Courses included in assessing GPT-4 capability on graduate examinations in the Biomedical Sciences. Columns include: Course: name of University of Florida (UF) graduate course; Exam Type: whether the examination was a course final exam (Final) or an end-of-year exam administered to first-year PhD students in the UF Genetics and Genomics Program (first-year); Blinding: whether GPT-4 answers were graded in parallel with student examinations using blinded identifiers (Full), whether grading was performed with knowledge of which exams were generated by GPT-4 (None), or a mixture of both types (Partial); Access: whether GPT-4 was queried using ChatGPT (ChatGPT) or a script accessing the OpenAI API (GPT-4 API); Figures: whether the exam questions contained graphical figures; Format: whether GPT-4 exam answers were handwritten (Paper) or digitally copied (Text) into the respective exam document; Simple/Expert/Short: Whether the GPT4-Simple, GPT4-Expert, and GPT4-Short prompt patterns was evaluated for each course’s examination, respectively.

### GPT-4 examination performance

**Figure 2:**
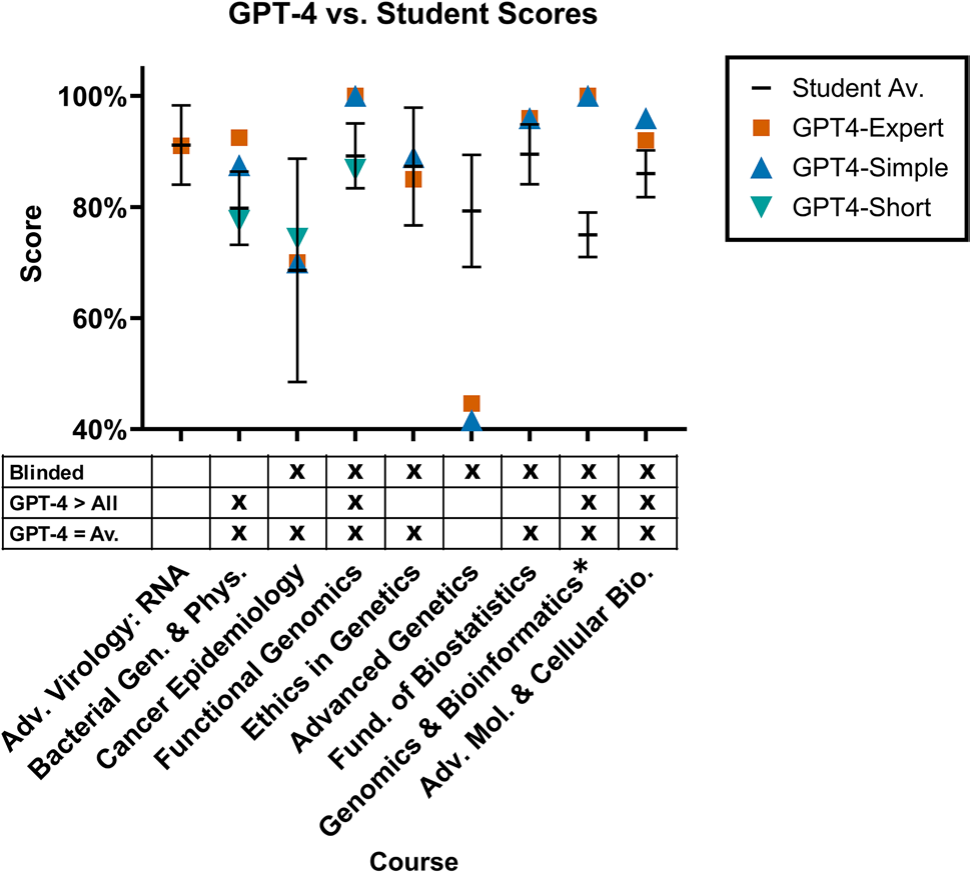
GPT-4 Performance on 9 graduate examinations in the biomedical sciences. GPT-4 using zero-shot or persona prompt patterns met or exceeded the average performance of graduate students in 7 courses in the biomedical sciences. The horizontal bar represents the student average score in the course and ranges represent sample standard deviation in student score values. In 7/9 cases, either the GPT4-Simple or GPT4-Expert prompt pattern met or exceeded average student performance in the course. Table Legend—Blinded: All GPT-4 exam grading was performed blinded in parallel with student assessments; GPT-4 > All: One or more GPT-4 scores exceeded all student scores; GPT-4 ≥ Av.: One or more GPT-4 scores exceeded the average student score. *GPT4-Simple and GPT4-Expert prompt patterns both received initial grades of 100% and final grades of 0% for the genomics and bioinformatics course based on suspected plagiarism due to similarities between answers.

Comparing individual GPT-4 course grades to student performance, GPT-4 answers scored at or above the average student score for 7 of 9 examinations (77%) exceeding performance of all students in 4 of 9 cases (44%), as shown in Fig. 2 and Supplementary Table 1. For GMS6231 (Genomics and Bioinformatics), the two GPT-4-generated answer sets both initially received scores of 100% but were marked as plagiarism based on a high degree of similarity between answers (with a final score of 0%). Details regarding specific context and scores for each examination are provided in the Supplementary Data.

Overall, mean GPT-4 performance across all courses (using initial scores for GMS6231) slightly exceeded student course average for each approach (+0.3% to +3.2%), though these differences were not statistically significant (*p* = 0.62 to *p* = 0.92) as shown in Supplementary Table 1. Performances between the GPT4-Simple and GPT4-Expert prompt patterns were also not significantly different with GPT4-Simple scoring higher than GPT4-Expert in 2/8 cases where both patterns were used (N = 8, *p* = 1.0). Likewise, GPT4-Simple exceeded the score of GPT4-Short in 2/3 cases, but the difference was not statistically significant (N = 3, *p* = 0.36).

### Performance on questions containing figures

While most examinations consisted entirely of textual questions, two examinations contained questions with figures, which were translated into text for input to GPT-4 as described in Methods. For each of these exams, GPT-4 performance was below the student average. In GMS6035 (Advanced Virology II: RNA Viruses) GPT-Expert scored −0.14% lower than the student average and in PCB5065 (Advanced Genetics) GPT4-Simple and GPT4-Expert scored −37.65% and −34.65% lower than the student average, respectively (Supplementary Table 1). To examine whether figure-including examinations corresponded to a significantly altered model performance, we separated examinations into those with and without figures. Group averages of GPT-4 score differences (difference between student average and mean GPT-4 score) with one or more figures in questions (N = 2, −18.14% ± 25.47% vs. student average [mean ± stdev.]) and no-figure (N = 7, +7.79% ± 8.08% vs. student average) were compared and the difference between groups was not statistically significant (*p* = 0.38, variance ratio 9.93).

To examine whether the GPT-4 knowledgebase contained information on specific published scientific figures, a set of exploratory queries were performed. One question that received full marks by GPT4-Expert required interpretation of a figure from a 2016 study by Bruner et al. regarding defective HIV proviruses (Bruner et al. Fig. 1, panels c and d)^46^. When ChatGPT (GPT-4) was asked to provide a description of this figure, it instead indicated that it does not have access to this information but provided a guess as to the expected content that closely matched the actual figure (Supplementary Fig. 2). Interestingly, further repeated queries using the GPT-4 API provided a mixture of similar denials that the model could provide this information (N = 15/25) interspersed with distinct, highly detailed hallucinated descriptions of a fictional figure ascribed to the publication (N = 10/25) which were often accompanied by interpretations of the significance of the fictional results (example in Supplementary Data).

### Survey results

Following grading, instructors for participating courses were provided perception surveys with free-text questions regarding the impact of GPT-4 in education, summarized in Supplementary Fig. 3. Most respondents indicated surprise at the quality of GPT-4 answers (N = 7/13) and a significant majority expressed concern that LLMs would be used by students for generation of answers even if prohibited (N = 10/13). Responses were mixed regarding expectations for future impact of GPT-4 on student learning, with respondents suggesting that GPT-4 could be an effective factual resource or tool for text summarization, while also raising concerns that use of the tool could impede development of students’ creativity, writing ability, and critical-thinking skills.

## Discussion

The recent emergence of capable chatbots such as ChatGPT has led to the rapid adoption of AI text-generation capabilities in many fields and has already begun shifting paradigms in scientific education. The convenient accessibility of GPT-4 and other LLM models now allows individuals from a broad range of backgrounds to access language-based AI tools without previous experience in the field. To both students and professionals in the biomedical sciences (as well as many other knowledge domains), the possibility of an expert “answer engine” that can clearly and correctly answer scientific questions is quite alluring. However, given that LLM models can compellingly present incorrect information and do not guarantee correct answers, the abilities and trustworthiness of these new methods need to be extensively validated before wide adoption for this purpose.

In our study we find that GPT-4 performs comparably to an above-average or exceptional graduate student on examinations in the biomedical sciences. GPT-4 excelled at textual short answer and fill-in-the-blank questions and received the highest marks for multiple essay questions. However, we also find GPT-4 performs poorly on questions based on figures with simulated data and in providing instructions for questions requiring a hand-drawn answer. During exploration of the knowledgebase of GPT-4, we additionally observe instances of detailed model hallucinations of scientific figures with realistic summative interpretation of these results. Together, these results demonstrate the capability and potential for use of LLMs as a tool in the sciences, but also exemplify the need for caution when using the current iterations of GPT-4 and ChatGPT for generation of text containing factual statements and interpretations.

### GPT-4 examination performance

For most exams, one or more GPT-4 prompt patterns met the average of students in the course in 7 of 9 examinations and exceeded all student grades in 4 of 9 cases. GPT-4 performance was not uniformly high, however, as GPT-4 answers scored poorly in an examination that incorporated extensive interpretation of scientific figures of simulated data and drawings with scores of 41.6% for GPT4-Simple and 44.6% for GPT4-Expert compared to 79.3% ± 10.10% for students. As ChatGPT & GPT-4 allowed only textual input during the period of data collection (March–May 2023), this result presents a potential limitation in our approach of providing the model with uninterpreted figure descriptions (as in Supplementary Fig. 4) and the model accuracy may be increased with figures described in domain-specific or technical language as would likely be found in relevant training data. With the recent expanded availability of image-based inputs to GPT-4 (and ChatGPT), current and future model versions may have a greater capacity to answer questions containing scientific figures using direct input of figure images to the model which should be studied in future work^45^.

GPT-4 did not struggle with all figure-containing questions, however. The Advanced Virology exam contained 7 of 13 questions based on interpretation of figures and GPT4-Expert received a score of 91.0% on this exam, nearly meeting the student average of 91.14%. One potential difference in this result compared to the Advanced Genetics exam is that labels included with figures from the Advanced Virology exam happened to be more verbose, including the full figure caption in one instance, which provided GPT-4 with more content for interpretation of questions. Further, figures in Advanced Virology questions were selected from published journal articles which may have been included in the model’s training data. For example, one question refers to Fig. 1c and d of a 2016 study by Bruner et al. regarding defective HIV proviruses^46^, with a textual description of this figure prepared and provided to ChatGPT along with the question text (Supplementary Fig. 4). For this question, GPT4-Expert received full marks. While the GPT-4 knowledgebase did not appear to contain the specific figure data, it did provide a close guess of figure content in our exploratory queries (Supplementary Fig. 2).

### GPT-4 hallucinations in scientific figure interpretation

Our exploration of model knowledge of scientific figures also provided an interesting example of model hallucinations. Using the GPT4-Expert persona pattern to request a description of the Bruner et al. figure initially resulted in a compelling hallucination of a fictional figure (Supplementary Fig. 2, Supplementary Data). On 24 further repeated trials of the same query, 9 additional responses contained similar descriptions of hallucinated figures (with differing content) attributed to Bruner et al., while the remainder either contained requests for a description of the figure or a denial that the model could provide that information. This variation in response to a factual query highlights the stochastic nature of LLM responses and exemplifies the risk in relying on current language models as sources of information. The rate of such hallucinations may be related to the model “temperature” parameter, which directly influences the randomness in model responses^12^. As this could play an important role in both response correctness and in detection of LLM-generated examination answers, the effect of this parameter on the accuracy of outputs to factual questions should be studied in future work.

### Influence of query prompt patterns on GPT-4 grades

As the content and style of GPT responses can depend upon specific query instructions, we compared the impact of two different prompt patterns on assessment scores. These were a “zero-shot” prompt pattern (GPT4-Simple) and two variants of the “persona” pattern (GPT4-Expert & GPT4-Short)^47^ as shown in Fig. 1. Sequential examination questions were also asked where possible within the same GPT-4 context window, implicitly following a “chain-of-thought” prompt pattern in which the answers to previous questions may influence the response to the next question^48^. While this strategy was employed to optimize GPT-4 performance across the whole of each respective examination (and mimic a potential real-world use case), future work could compare performance when GPT-4 is provided individual examination questions in independent query sessions.

Contrary to our expectations, the prompt pattern used for querying GPT-4 did not significantly impact answer grades. The GPT4-Simple pattern, which provided a minimal preface to questions, received similar scores to the GPT4-Expert prompt which provided extensive instructions to act as an expert in the field, including formatting instructions and instructions on answer length (full prompts in Supplementary Methods). Qualitatively, although answers between these methods differed in format as per prompt instructions, they often subjectively appeared as highly similar in content. In an analysis of the Flesch-Kincaid Grade Level of responses to an example question, both methods provided answers at a post-secondary level with GPT4-Simple at 15.1 and GPT4-Expert at 18.6 (shown in Supplementary Data)^49^. These results may imply promise in future use of this model as an “answer engine,” suggesting that the model’s scientific answer accuracy would not be highly sensitive as to whether the model is instructed to act as an “expert.” However, an alternative possibility is that responses were influenced in this case by the expert-level wording in the specific text of the examination questions, which may induce a particular type (and perhaps accuracy) of response. As such, further characterization is needed to assess trustworthiness of answers over several domains with repeated queries before adoption for this purpose.

The GPT4-Short approach was developed in response to our initial observations that GPT-4 answers were more verbose than would be provided by a student, even when provided specific instructions for answer length and brevity as with the GPT4-Expert prompt. As such, we developed the strategy of using a separate instance of the GPT4-Expert prompt pattern but then iteratively directing GPT-4 to shorten every answer to approximately 65% of the initial length (Fig. 1). Qualitatively, this approach resulted in answers more closely matching student answer length but often resulted in the loss of potentially important details from answers rather than inducing an increase in “terseness” which might be observed from a student trying to write efficiently in a limited time. Additionally, this process did not significantly decrease the Flesch-Kincaid Grade Level in an example answer instance, with scores of 18.6 GPT4-Expert and 16.1 for GPT4-Short as shown in the Supplementary Data. In the Cancer Epidemiology course, GPT4-Simple and GPT4-Expert answers appeared especially verbose and GPT4-Short received a higher score in comparison: (GPT4-Short: 74.3% vs. GPT4-Simple: 70.0% and GPT4-Expert: 70.0%). However, in two other courses GPT4-Short received a ~13% lower grade than the average of GPT4-Simple and GPT4-Expert, with instructor comments indicating missing details from several answers that were not noted in the corresponding grades for GPT4-Expert.

### Use of ChatGPT in higher education

From our observations, ChatGPT has been increasingly adopted among both academic faculty and students. Faculty use ChatGPT for generation of presentation titles, slide content, exam questions, and writing reference letters. Students extensively use ChatGPT as a reference resource to create practice quizzes, generate digital flashcards, create mnemonics for memorization, source anecdotal information, and summarize journal articles or figures. However, we also observe students utilizing ChatGPT for assignment tasks such as writing essays and research papers, summarizing journal articles, performing statistical analyses, and generating solutions to programming problems^50^. After unblinding of GPT-4 results, a majority of instructors responding to surveys indicated both surprise at the quality of GPT-4 answers to expert-level scientific questions and concern for potential student use of LLMs (Supplementary Fig. 3). As student reliance on generative text methods to complete assignments may impede development of necessary critical-thinking, problem-solving, and writing skills, academic training and assessment methods will need to adapt to preserve efficacy and integrity in light of the performance capabilities of the GPT-4 models evidenced in this study (as well as future, improved LLM models).

### Trends in GPT-4 answers

In addition to providing an assessment of GPT-4’s capabilities, our results provide insights for modification of student assessments as LLM chatbots continue to increase in capability and accessibility. The UF Genetics and Genomics Graduate Program requires all first-year PhD students to pass end-of-year comprehensive examinations for the five required first-year courses (Advanced Genetics, Introduction to Biostatistics, Ethics in Genetics, Genomics and Bioinformatics, and Advanced Molecular and Cellular Biology). These exams are written and graded by instructors of the first-year courses. In May 2023 after grading two GPT-4-generated exam answers in addition to student exams, the course instructors met with program administrators to unblind and discuss the results and to brainstorm ways to “GPT-proof” future exams.

Before unblinding, most instructors correctly guessed which answers were GPT-generated. Instructors noted several trends differentiating GPT-4 answers from student answers:

- GPT-4 answers to questions that required the interpretation of experimental data were long, excessively wordy, and often included accurate but un-requested information and/or explained simple ideas that were obvious but not required for the answer, e.g. description of a box plot or an ANOVA test.
- In a multi-paragraph essay answer, different GPT-4 answer-sets used distinctively similar organization and wording, e.g. the first paragraph started with “In this study, …” and the final paragraph started with “In conclusion/summary…”. These differences presented a clear distinction to instructors between GPT-4 and student answers.
- GPT-4 answers to questions that required interpretation of simulated experimental data uniformly received the worst grades, e.g. “0—no clue”.
- Conversely, GPT-4 answers to a basic knowledge essay question “Explain how the nucleotide diversity measured in a population is related to its effective size and the mutation rate of the species” received perfect scores.
- GPT-4 answers to questions that required students to draw or interpret a diagram or make calculations based on a diagram with simulated data generally received the lowest scores.
- GPT-4 answers to short-answer questions were 100% correct.
- GPT-4 answers to multi-step statistical questions were 100% correct. The instructor thought the inclusion of multiple steps and short questions that referred to information given only in previous questions would make it harder for GPT-4 to give correct answers, but that assumption was incorrect.
- In two out of five essay questions, GPT-4 answers received the highest grades.
- GPT-4 answers to essay questions that required students to include and refer to specific course materials had lower scores than student answers.
- Answers to the ethics essay question resulted in good grades (B and B+), but were assessed by the grading professor as generic and bland or lacking in conviction. They often did not state a clear position (even though taking a position was part of the question) and lacked explicit reasoning, specific examples to support points, and general follow-through on points made.

The Genomics and Bioinformatics essay question for which GPT-4 answers were flagged as plagiarism referenced a specific method (Perturb-seq and single-cell proteomics) and a related journal article, asking students to explain the approach, limitations, and recent advances. In general, GPT-4 answers for essay questions were sufficiently different that instructors did not think they were written by the same person—the more explicit instructions in the questions in the plagiarism case may have induced GPT-4 answers to adopt a more similar wording and organization of answers. Interestingly, a similar question that referenced a specific method and related journal article, but also included the article abstract, resulted in GPT-4 answers that received the highest grades for the question without concerns of plagiarism.

### Modification of examinations for the Chatbot era

Given the significant capability of LLM models to answer logic and reasoning questions for biomedical science exams observed in our study, we are adopting a set of recommendations for adapting assessments for our future courses. Importantly, many of our observations on answer format, length, and style discussed above are based on the grading of *unedited* answers provided by GPT-4. As with other non-original sources of essays and examination answers, a sufficiently motivated student could use an initial draft of an answer by ChatGPT and adapt this answer to match their own voice and fit the requested length^50^. Further, actionable suspicions of plagiarism require direct evidence and definitive detection of LLM content remains a major challenge^51,52^. Thus, for recommendations to make questions more resistant to GPT for our courses, we are focusing on systematic barriers and question design rather than on identification of patterns and formatting. For in-person and remote-learning examinations, we believe use of “no-device” policies and/or secured testing environments should prevent LLM access similarly to other prohibited information sources. For take-home examinations and essay assignments, we are adopting the following recommendations:

- Write questions that include complex figures based on simulated data that must be interpreted or used as part of the answer (GPT-4 scored poorly in our study on questions based on simulated data presented as figures).
- Write questions that require students to draw a figure as part of the answer.
- Avoid short-answer questions or questions that query basic, elementary knowledge that is easily found on the internet.

In addition to these suggestions, we considered other means by which LLM-methods could be circumvented or detected if used by students for generation of answers. However, upon further discussion we identified some means that each of these could be overcome (listed below):

- For essay questions, require students to refer to multiple course materials, such as lecture slides.
  o This strategy may be countered by students copy/pasting the relevant course material text into ChatGPT, or by using a programming script to feed course slides as background for the question into the OpenAI API.
- Require answers to refer to specific references to published journal articles.
  o Students could iteratively direct ChatGPT to include information from specific references by copying reference abstracts or paragraphs as required.

### GPT-4 as a reference resource

While generative text methods seem likely to become an integral tool in many facets of the future, it is important to evaluate capabilities of LLMs across many topics and response types before relying on these new tools for accurate information. This is especially true for use of LLM’s in education or as a reference resource, as entirely false responses can be near-indistinguishable from fact. The high accuracy that we observe in GPT-4 responses to graduate-level examination questions demonstrate the model’s capability to generate correct answers to many expert-level scientific questions. However, GPT-4 performed poorly on questions with textual figure descriptions of simulated data and we observed several cases of detailed GPT-4 hallucinations when asked about a scientific resource. This overall high level of GPT-4 examination performance also indicates that the format of many scientific exams may need to adapt to decrease the temptation for students to illicitly turn to this easily accessible resource. As such, while we suggest that users should not rely solely on current LLM’s like GPT-4 as sources of reference information, such models may soon function as convenient and reliable expert resources across the sciences.

## Methods

### Included courses and examination format

Course instructors from 9 graduate courses in the biomedical sciences at the University of Florida (UF) agreed to participate in the study after recruitment via email. Examinations included in the study were administered from March to May 2023 to course students either in person on a paper form, via a secure web-based test administration system, or via distribution of a textual document in which answers were filled by the student in an unsecured environment. Examination questions were primarily in the style of short response (1/4- to 1/2-page), also including fill-in-the-blank, essay, and diagram-drawing questions. A few questions included a scientific figure or diagram as Supporting information.

### GPT-4 query method and formatting

Question answers for one examination (GMS6035: Advanced Virology II: RNA Viruses final examination) were generated via interactive usage of GPT-4 via ChatGPT (https://chat.openai.com). For the remaining courses, the OpenAI API was used to query GPT-4 using a custom script written in Python3.8 with the OpenAI python module. Queries for examination questions were made to the “gpt-4” model between 2023-05-06 and 2023-06-16 using openai.ChatCompletion.create(model=’gpt-4’, prompt=[prompts]) from the OpenAI python module with default values for other call parameters. This script and example outputs are provided via GitHub (see Data and Availability). Answers for each combination of examination and prompt pattern were generated via independent chat sessions with ChatGPT or independent access sessions to the OpenAI API.

For exams delivered on paper, exam questions were transcribed into textual form by a member of the study staff. For exams distributed digitally, examination question content was copied from the source textual document and converted to plaintext for entry into the relevant query method. Where a question did not specify the expected length of the answer, the length of the expected answer (1/2-page, 1/3-page, etc.) was determined by the blank space following the question on the relevant exam form and an annotation was placed next to the question identifier to indicate the expected answer length in queries to GPT-4.

As multimodal image input for GPT-4 was not publicly available at the time of the study, questions containing figures were modified by replacement of each figure with a corresponding textual description in the question. To avoid the addition of bias by the study staff, figures were not interpreted and were described by creating a textual description of each shape, color, label, number, and caption in the figure and their relative positions to each other. Any provided figure textual descriptions were also included. An example version of the result of this process is shown in Supplementary Fig. 4.

### GPT-4 prompt patterns

The content and style of responses provided by GPT models can be influenced by the specific instructions provided in a query. Thus, we compared the use of three variations of two different instruction styles (prompt patterns) in parallel to observe their potential impact on assessment scores. The evaluated variants were: “GPT4-Simple:” a “zero-shot” pattern in which each question was directly asked to GPT-4 without specific instructions as to the format of the examination answer; “GPT4-Expert:”, a persona pattern in which GPT-4 was instructed to answer as an expert in the knowledge domain of the test with specific instructions on answer formatting; and “GPT4-Short”, a persona pattern including the same instructions as GPT4-Expert but with subsequent instructions in a follow-up query to shorten each answer by 65%^47,48^. Sequential examination questions were asked where possible within the same GPT-4 context window, also implicitly following a “chain-of-thought” prompt pattern where answers to previous questions may influence the response to the current question. An example of each utilized prompt pattern is provided in the Supplementary Methods.

### GPT-4 answer formatting and grading

Following generation of GPT-4 answers, GPT-4 responses were copied into the relevant document following the examination format. As members of the study staff have significant experience in topics of examination in this study, our procedures were designed to minimize addition of bias to GPT-4 responses by exactly copying GPT-4 answers into examination forms. Where responses included a hand-drawn component, a member of the study staff drew a diagram following the instructions provided in the GPT-4 response as closely as possible with the goal of excluding their own relevant background knowledge, with any respective answers to the same question from different approaches (GPT4-Simple, etc.) drawn by a different member of the study staff.

For all examinations, answers generated by GPT-4 were either handwritten into the examination form or copied into the appropriate space to match the style of student answers. Any hand-drawn diagrams for answers were photographed and included as an image in the answer form to match the style of answers provided by students. Answer sources were blinded to grading professors where possible given logistic constraints, with grading of seven examinations (and part of another) performed in parallel to the respective course students using blinded identifiers. Blinding of exams are described in Table 1 with specific details regarding exam logistics provided in the Supplementary Data.

### Acquisition of student performance data

For collection of student performance information on included examinations, the study was determined as exempt research by the University of Florida Institutional Review Board protocol IRB202301291 and no informed consent was required or collected. All relevant methods were carried out in accordance with relevant guidelines and regulations. Anonymous student grade information was then collected from course directors corresponding to exams completed by GPT-4.

### Conversion of letter grades to numeric grades

Where required, letter grades assigned to student and GPT-4 examinations were converted to numeric grades by selecting the middle score of the range traditionally used by the study staff for grade assignment (rounded up to the nearest integer). Rubric: “A+/A”: 96, “A−”: 92, “B+”: 89, “B”: 85, “B−”: 82, “C+”: 79, “C”: 76, “C−”: 72.

### Statistical analysis

Analysis of grade data was performed in Microsoft Excel 365. The arithmetic mean of sets of grade values were calculated with =AVERAGE() and sample standard deviations were calculated with =STDEV.S(). For comparison of student grade averages to GPT-4 scores and between sets of GPT-4 scores, two-tailed paired T-tests were performed with =T.TEST([Range1], [Range2], 2, 1). Score differences (“GPT4-Expert Diff,” etc.) were calculated by subtracting the student mean exam score from the respective GPT-4 score. For comparison of average GPT-4 grades between the with-figure and without-figure conditions, the mean GPT-4 score difference for each exam was first calculated using =AVERAGE() across GPT-4 score difference values for each respective exam. The sample variance in sets of means was then calculated with =VAR.S() for each condition and the variance ratio between sets was calculated with =([Var1]/[Var2]). For comparison between conditions, the two-tailed t-test with unequal variance was selected and calculated using =T.TEST([Range1], [Range2], 2, 3) function.

### Surveys

For collection of instructor opinions, a survey instrument was designed and determined as exempt research by the University of Florida Institutional Review Board protocol ET00018705 and no informed consent was required or collected. All relevant methods were carried out in accordance with relevant guidelines and regulations. This instrument was supplied to course instructors following grading and any unblinding of GPT-4 and student results. The survey instrument was designed as a series of free-text response questions. For summarization of answers to questions presented in Supplementary Fig. 3, the main theme of each response was evaluated by study staff. If a response was determined as clearly falling into a category such as “yes vs. no,” or “positive vs. negative,” it was tallied as such or was otherwise marked as “indeterminate” or “other” depending on the specific question.

### Exploratory ChatGPT and GPT-4 queries

Exploratory Queries to GPT-4 via ChatGPT as shown in Supplementary Fig. 1 and Supplementary Fig. 2 were performed between 2023-06-27 and 2023-07-11 as indicated in each respective figure via chat.openai.com. For exploratory queries of model knowledge of figures performed via the OpenAI API, the “gpt-4-0314” model was selected to mirror the model used in in the Advanced Virology exam questions. Queries were performed via a custom script between 2023-07-16 and 2023-08-25 using the procedure described above (see Data availability).

### Example GPT-4 answers for each prompt pattern

For the sample formatted exam question provided in the Supplementary Data, example answers for each prompt were generated via access to the OpenAI API “gpt-4” model via a custom script on 2023-07-16 using the procedure described above (see Data Availability). The Flesch-Kincaid Grade Level for each response was calculated using Microsoft Word 365. Each response was copied into an independent document and the Review (Tab) → Editor (Button) → Document stats (Button) tool was used to calculate the grade level of the response.

### Manuscript preparation

Manuscript figures were created using Graphpad Prism and Microsoft PowerPoint using colors selected from the Bang Wong colorblind-friendly color scheme^53^. Textual editing was performed using Microsoft Word and EndNote. No LLM’s or Chatbots were used in the preparation of the text of this manuscript.

### Supplementary Information

Supplementary Information.

## Data Availability

The Python3 script used for querying the OpenAI API and example outputs are available at http://github.com/dstrib/GPT4_Biomed_Assessment and via Zenodo^54^. Specific examination questions utilized in the study may be available upon request with the permission of the professor who provided the question. Individual student scores are omitted from available study data for student privacy. Other study data is available upon request to Daniel Stribling.
