# Supplementary material for: The model student: GPT-4 performance on graduate biomedical science exams
Source: Sci Rep. 2024 Mar 7;14:5670. doi: 10.1038/s41598-024-55568-7 (PMC10920673; doi:10.1038/s41598-024-55568-7)
Supplement: Supplementary file 1 — Supplementary Information. [file 41598_2024_55568_MOESM1_ESM.pdf]

The Model Student: GPT-4 Performance on Graduate Biomedical Science Exams: Supplementary Information

Daniel Stribling[1,2,3,*]; Yuxing Xia[4,†]; Maha K. Amer[1]; Kiley S. Graim[5]; Connie J. Mulligan[2,6]; Rolf Renne[1,2,3,*]
* Correspondence: ds@ufl.edu, rrenne@ufl.edu

1 Department of Molecular Genetics and Microbiology, University of Florida, Gainesville, FL 32610, USA.

2 UF Genetics Institute, University of Florida, Gainesville, FL 32610, USA.

3 UF Health Cancer Center, University of Florida, Gainesville, FL 32610, USA.

4 Center for Translational Research in Neurodegenerative Disease, Department of Neuroscience, College of Medicine, University of Florida, Gainesville, Florida, 32610, USA

5 Department of Computer & Information Science & Engineering, Herbert Wertheim College of Engineering, University of Florida, Gainesville, FL 32610, USA

6 Department of Anthropology, University of Florida, Gainesville, FL, 32610, USA

† Current Address: Department of Neurology, UCLA, Los Angeles, CA, 90095, USA.

Scientific Reports

DOI: 10.1038/s41598-024-55568-7 (https://doi.org/10.1038/s41598-024-55568-7)

Supplementary Methods:

Zero-shot prompt pattern (“GPT4-Simple”):

```
"""
----- system -----
Please answer the following questions.

 ----- user -----
I am ready to provide you with questions to answer.

 ----- assistant -----
Please provide the questions from the examination.
"""
```

Persona prompt pattern (“GPT4-Expert”):

```
"""
----- system -----
I am going to give you questions from an examination in a graduate course in Cell Biology.
Please act as an expert in the field of Cell Biology.
Please answer each question as correctly as possible, using technical or advanced language as
necessary to answer the question correctly.

Details regarding the examination:

Answers will be entered as plaintext into the response portion of a document as a student in
an exam setting, so do not include any markdown symbols.
Your answers should be extremely concise and extremely clear so that they can fit into the
answer textbox for the examination.

Some questions may have multiple parts, denoted by letters after the question number. For
example: 1A and 1B. When answering multiple part questions, refer to the answer of previous
parts of the question as necessary to answer each question correctly.

For each question, respond with an answer as a narrative paragraph, without including a list
in the answer.
If the answer to a question contains multiple ideas, components, or steps, respond with
narrative paragraphs connecting topics or ideas.
If a question asks you how you would do something, respond with a narrative paragraph and do
not separate the answer into a list of steps.

Some questions will refer to a figure, chart, or graphic. For these questions, ask for a
description of each panel of the graphic before answering the question, and then use the
graphic to answer the question as needed. **Do Not** answer questions that refer to a figure,
chart, or graphic without first asking for a description.

Some questions will request you to draw a figure or diagram to assist in answering the
question. This is signified by keywords: "draw" or "sketch" in the question.
For these questions, after providing the text of your answer, provide a full
page of extremely detailed drawing instructions to draw up to one graphic as appropriate to
answer the question. For drawing instructions, first start with the text: "[Drawing
Instructions]". Then, provide detailed instructions on each shape and line to be drawn, and
their relative position to the other shapes and lines in the drawing. Then provide any
captions to be drawn as well as indicating what shapes or lines should be captioned.

If a question refers to materials, sources, or references used in the course, do your best to
guess what materials were used in the course to answer the question.
Provide citations for sources if the question asks for sources or references.

Remember, answer all questions in narrative form, acting as an expert in the field.
Answers should be extremely clear and extremely concise.
```

```
Thank you!

 ----- user -----
I am ready to provide you with questions to answer.

 ----- assistant -----
Please provide the questions from the graduate-level examination. I will provide extremely
clear and concise answers to each question, acting as an expert in the field. My answers will
be provided as narrative paragraphs and will not include a list unless the question
specifically asks for a list. If the question mentions a figure, chart, or graphic, I will ask
for a description of the figure, chart, or graphic before answering the question. If the
question mentions providing a drawing or sketch, I will provide detailed instructions on how
to draw a graphic illustrating my answer after providing the text of my answer. Please provide
the first question.
"""
```

In the case of PHC7007: Cancer Epidemiology, an additional instruction was provided after the question response to remove numeric lists if the string: “`1. `” was detected in model outputs:

```
"""
The last answer you provided included a list. Please restate the answer as paragraphs of
clear, concise narrative text without any numeric lists.
"""
```

Shortened persona prompt pattern (“GPT4-Short”):
[Initial three prompts as with “GPT4-Expert” prompt pattern]

```
"""
----- assistant -----
[…initial response…]

 ----- user -----
Please shorten the last answer to approximately sixty-five percent of the original length.
The shortened answer should be correct, clear, and concise without any numeric lists.
"""
```

- Exploratory ChatGPT-Based GPT-4 Querying with Partially Blinded Assessment for Final Exams

Student answers for the GMS6035: Advanced Virology II: RNA Viruses final examination were handwritten by students (N = 7) in a University of Florida classroom setting on an examination paper with answers graded by two professors. To explore the possibility of use of ChatGPT to create answers suitable for handwriting on an examination form in this fashion, a persona pattern was used providing instructions as to answer length and format as shown in **Supplementary Fig. 5a**. Answers for the first section of this exam were generated interactively using ChatGPT, providing intermediate additional instructions as to answer length and formatting (but not content) throughout the process (**Supplementary Fig. 5b**). To better match answers that would be handwritten by students, ChatGPT was later instructed to highlight important concepts using bold text, which were handwritten using underlines during transcription of answers to physical paper. Grading of this portion of the examination occurred unblinded to solicit developmental feedback on abnormalities in question answer format in comparison to those traditionally provided by students. The average student grade for this section (grade

± std. dev.; of 60 possible points) was 56.43 ± 3.41 (94.0% ± 5.7%), whereas GPT-4 with the initial "Expert" persona received a grade of 55 (91.7%).

Based on the results of the first part of the exam, an updated version of the initial expert persona pattern was used (**Supplementary Fig. 6**). Answers for the second portion of this exam were generated interactively using ChatGPT with intermediate additional instructions as to answer length as with Part I, but no additional instructions were given as to formatting. Answers provided by ChatGPT were handwritten onto an examination form and grading was performed using blinded identifiers in parallel with student assessments. The average student grade (of 40 possible points) for this section was 34.71 ± 5.59 (86.8% ± 13.98%), whereas GPT-4 with a second trial version of the "Expert" persona received a grade of 36 (90.0%). Combining both portions of the exam, the overall student score (out of 100 possible points) was 91.1 ± 7.15 (91.1% ± 7.15%), with a GPT-4 "Expert" persona grade of 91.0 (91.0%).

- API-Based GPT-4 Querying with Unblinded Assessment for Final Exams

Students in the GMS6038: Bacterial Genetics & Physiology course (N = 8) were administered the course final examination via an online testing system. Examination questions were provided to GPT-4 via a custom script using the OpenAI API (see **Methods**) and answers were generated using the GPT4-Simple, GPT4-Expert, and GPT4-Short approaches and copied into the relevant portion of the examination form. At the time of the study, student grading for this examination had already occurred and as such GPT-4 generated answers for each pattern were assessed by the grading professor unblinded to answer source, but without knowledge of prompt pattern used for each answer set. The average student grade (out of 100 possible points) was 79.8 ± 6.6 (79.8% ± 6.6%). GPT-4 received grades of: GPT4-Simple: 87.5 (87.5%), GPT4-Expert: 92.5 (92.5%), and GPT4-Short: 77.5 (77.5%), with the GPT4-Simple and GPT4-Expert scores exceeding the student average.

- API-Based GPT-4 Querying with Blinded Assessment for Final Exams

Students were administered the GMS6473: Fund. of Physiology & Functional Genomics (N = 2) and PHC7007: Cancer Epidemiology final examinations (N = 4), respectively, via submission of a textual document. For each exam, GPT-4 was accessed via a custom script using the OpenAI API and answers were generated using the GPT4-Simple, GPT4-Expert, and GPT4-Short prompt patterns as described in **Methods**. For both exams, grading of GPT-4 answers was performed using blinded identifiers in parallel with student assessments.

For GMS6473: Fund. of Physiology & Functional Genomics, only part of the examination was able to be included in the study due to logistic considerations, representing 60% of the total available points, with student and GPT-4 percent results for this exam given of these 60 points. The average student grade (out of 60 included points) was 53.5 ± 3.5 (89.2% ± 5.8%). GPT-4 received grades of: GPT4-Simple: 60.0 (100%), GPT4-Expert: 60.0 (100%), and GPT4-Short: 52 (86.7%), with the GPT4-Simple and GPT4-Expert scores exceeding all student grades.

For PHC7007: Cancer Epidemiology, a modified version of the GPT4-Expert strategy was used in which a follow-up prompt requested the removal of numeric lists when these were included in the GPT-4 answer (prompt provided in **Supplementary**

Methods). The average student grade (out of 70 possible points) was 48.0 ± 14.1 (68.6% ± 20.1%). GPT-4 received grades of: GPT4-Simple: 49.0 (70.0%), GPT4-Expert: 49.0 (70.0%), and GPT4-Short: 52.0 (77.5%), with all GPT-4 scores exceeding the student average.

- API-Based GPT-4 Querying with Blinded Assessment for Year-End Exams

First-year students in the UF Genetics & Genomics PhD Program complete the following courses over their initial fall and spring semesters: GMS6231: Genomics and Bioinformatics; PCB5065: Advanced Genetics; BCH6415: Advanced Molecular and Cell Biology; PHC6052: Introduction to Biostatistical Methods; and GMS6221: Ethics in Genetics, followed by first-year examinations at the end of the spring semester. First-year Genetics & Genomics PhD students (N = 4) completed first year examinations for the above courses administered via distribution of textual documents in an unsecured environment over a 5-day period. For each of these examinations, GPT-4 was accessed via a custom script using the OpenAI API and answers were generated using the GPT4-Simple and GPT4-Expert prompt patterns (see **Methods**) and copied into the respective examination answer forms. Grading of GPT-4 answers was then performed using blinded identifiers in parallel with student assessments. For the PHC6052, BCH6415, and PHC6052 courses, grades were reported as letter grades and these were converted to numeric grades as described in **Methods**.

For GMS6231: Genomics and Bioinformatics, the average student grade (out of 20 possible points) was 15 ± 0.8 (75.0% ± 4.0%). GPT4-Simple and GPT4-Expert answer sets initially received respective grades of 100% (surpassing all student grades) but were marked as suspiciously similar and were assigned a final grade of 0% for plagiarism.

For PCB5065: Advanced Genetics, the exam contained two questions with figures and one drawing question. The average student grade (out of 100 possible points) was 79.3 ± 10.1 (79.3% ± 10.1%). GPT-4 received grades of: GPT4-Simple: 41.6 (41.6%) and GPT4-Expert: 44.6 (44.6%), with all student scores exceeding all GPT-4 scores.

For BCH6415: Advanced Molecular and Cell Biology, student grades were converted from letter grades and the average student grade was 86.0% ± 4.2%. GPT-4 received grades of: GPT4-Simple: A+ (96%) and GPT4-Expert: A- (92%), with both GPT-4 methods exceeding the student average and GPT4-Simple exceeding the top student score.

For PHC6052: Introduction to Biostatistical Methods, student grades were converted from letter grades and the average student grade was 89.5% ± 5.4%. GPT-4 received grades of: GPT4-Simple: A (96%) and GPT4-Expert: A (96%), with both GPT-4 methods exceeding the student average score.

For GMS6221: Genetical Ethics, the first of two possible choices for question 1 was chosen for GPT-4 responses. Student grades were converted from letter grades and the average student grade was 87.3% ± 10.6%. GPT-4 received grades of: GPT4-Simple: B+ (89%) and GPT4-Expert: B (85%), with GPT4-Simple exceeding the student average score.

- Full GPT-4 Responses to a Question from Adv. Virology Exam Using Three Different Prompt Patterns

The following is a question from the GMS6035 Advanced Virology II: RNA Viruses final examination formatted as used in the OpenAI API model queries. The formatted question includes a textual description of a figure (Bruner et al., 2016, Figure 1, panels c and d)[1], describing the image of the actual figure used in the exam. GPT-4 responses using the OpenAI API (GPT-4, 2023-07-16) are provided for the GPT4-Simple, GPT4-Expert, and GPT4-Short prompt patterns as described above.

- - Formatted Question Text:

```
Q2) We read the paper Bruner et al. in which they characterize the latency reservoir of HIV in
cART treated individuals.
They found that only a small fraction (about 2%) actually harbor replication competent
proviruses. below is Figure 1 c and d depicting the genome structure of the proviruses they
analyzed by sequencing and a pie chart showing the mutations they found.
Please explain for **any four** of the eight color-coded categories in the pie chart d below
the mechanism(s) of how these mutations occurred, and how they would affect viral replication.
(12 points)
Figure description: Figure 1 c shows many horizontal colored rectangles with the labels: LTR,
gag, pol, vif, vpr, vpu, tat, rev, env, nef, and LTR. It also shows three sets of many
horizontal lines with different sections of these lines colored blue, green, white, and other
colors. The three groups of lines have the labels CP07, CP03, and CP10. The legend for line
colors contain the labels: Intact sequence, Inferred intact, Hypermutated, Inferred
Hypermutated, Not sequenced, min. Deletion, Mapped deletion, and Max Deletion.
Figure 1 d shows a pie chart depicting colors with the labels:
2% Intact,
5% Packaging signal and major splice donor site deletion
20% Deletion within 5' half of the genome
35% Deletion within 3' half of the genome
20% Very large internal deletion
7% Hypermutated
8% Hypermutated and deleted
3% Sequence insertions or inversions
The pie chart has the caption: n = 10 individuals, 152 sequences.
```

- - GPT4-Simple Response:
Flesch-Kincaid Grade Level: 15.1

```
Based on the given information from the pie chart in Figure 1d:

1) 5% Packaging signal and major splice donor site deletion:
Mechanism: This class of mutations is caused by the deletion of the packaging signal (psi) and
major splice donor (MSD) site in the viral genome.
Effect on replication: The packaging signal is responsible for the selective packaging of the
viral genome into virus particles. Without it, the viral RNAs cannot be incorporated into new
virions, rendering the virus non-infectious. The major splice donor site is essential for
splicing viral mRNAs into different isoforms, which are crucial for the proper expression of
viral genes. If this site is deleted, the proper splicing of viral mRNAs is disrupted,
impairing the synthesis of protein products and thus the replication capacity of the virus.

2) 20% Deletion within 5' half of the genome:
Mechanism: Occurs through recombination events, non-homologous end joining, or transcription
errors, resulting in a deletion in the 5' half of the viral genome that includes genes such as
gag and pol.
Effect on replication: Deletions in the 5' half of the genome disrupt the functions of key
genes involved in viral replication, such as gag (capsid assembly) and pol (reverse
transcriptase, integrase, and protease). The loss of these essential genes hampers the virus's
ability to replicate and produce infectious virions.
```

3) 35% Deletion within 3' half of the genome:
Mechanism: Like the previous mechanism, deletions within the 3' half of the genome can occur through non-homologous end joining, recombination events, or transcription errors.
Effect on replication: Deletions in the 3' half disrupt crucial genes, such as env and the accessory genes (vif, vpr, vpu, tat, rev, and nef), that play important roles in the viral life cycle. In particular, alterations or deletions in the env gene affect the viral envelope glycoproteins, which are responsible for the recognition and fusion of the virus with the target host cell. Additionally, the accessory genes contribute to many aspects of the viral life cycle, immune evasion, and pathogenesis. Deletions affecting these genes compromise the virus's ability to effectively infect new cells.

4) 7% Hypermutated:
Mechanism: Hypermutation occurs as a result of host restriction factors, such as APOBEC3G, that cause extensive G-to-A or C-to-T mutations in the viral genome during reverse transcription.
Effect on replication: Hypermutation introduces numerous stop codons and missense mutations in the viral genes, leading to the production of truncated and nonfunctional proteins. This prevents the virus from completing its replication cycle and producing infectious progeny.

- - GPT4-Expert Response:
Flesch-Kincaid Grade Level: 18.6

I will address the mutation categories for 1) Packaging signal and major splice donor site deletion, 2) Deletion within the 5' half of the genome, 3) Hypermutated, and 4) Hypermutated and deleted mutations from the pie chart in Figure 1d and discuss their effect on viral replication.

1) Packaging signal and major splice donor site deletion (5%): These mutations occur when the HIV genome undergoes recombination, either through homologous recombination between two similar sequences or illegitimate recombination due to error-prone DNA repair. The packaging signal is essential for incorporating the HIV RNA genome into the viral particles. Deletion of the packaging signal could prevent the virus from packaging its RNA genome, thereby inhibiting the formation of infectious virions. Similarly, deletions in the major splice donor site impair mRNA splicing, reducing the production of necessary viral proteins and resulting in nonfunctional or noninfectious virions.

2) Deletion within the 5' half of the genome (20%): These deletion mutations occur through mechanisms similar to those mentioned above (homologous or illegitimate recombination). Deletions in the 5' half of the genome could disrupt essential viral genes such as gag and pol, which encode structural proteins and enzymes required for viral replication. Consequently, these deletions would impair the proper assembly of viral particles and hinder the viral life cycle by disrupting reverse transcription, integration, and other essential processes.

3) Hypermutated (7%): Hypermutation occurs predominantly as a restriction mechanism against HIV-1, mainly by the cellular enzyme APOBEC3G. This enzyme deaminates cytosine residues to uracil in newly synthesized viral DNA, causing extensive G-to-A mutations when read by reverse transcriptase. These hypermutations can lead to the introduction of premature stop codons, frameshifts, and other detrimental changes in the coding sequences, severely affecting the functionality of viral proteins and essentially hindering viral replication.

4) Hypermutated and deleted (8%): This category represents proviruses that are both hypermutated, as explained in the previous point, and have deletions. Deletions may arise from recombination events, as discussed earlier. The combination of hypermutation and deletion events exacerbates the impact on viral replication by affecting multiple regions of the genome and inhibiting essential viral functions.

Overall, these mutation categories represent replication-competent proviruses in the HIV-1 latency reservoir in cART-treated individuals and can significantly impair the ability of HIV to produce infectious virions.

- - GPT4-Short Response:
Flesch-Kincaid Grade Level: 16.1

1. Packaging signal and major splice donor site deletion mutations result from erroneous reverse transcription and DNA repair. These mutations render proviruses replication-incompetent as they impact essential processes such as RNA packaging and splicing.

2. Deletions within the 5' half of the genome likely stem from flawed reverse transcription, integration, or DNA repair. These deletions disrupt essential viral genes, such as gag, pol, and vif, impairing the provirus's ability to encode necessary proteins for assembly and replication.

3. Deletions within the 3' half of the genome similarly arise from errors during reverse transcription, integration, or DNA repair. These deletions affect critical viral genes, including env, vpu, vpr, tat, and rev, which have vital roles in viral entry, immune evasion, and gene regulation, leading to replication incompetence.

4. Hypermutations in HIV proviruses are mainly caused by the host restriction factor APOBEC3G, which introduces excessive mutations during reverse transcription. These mutations often result in nonfunctional proteins, premature stop codons, and frameshifts, rendering the provirus replication-deficient.

- Hallucinated Responses by GPT-4

Querying the GPT-4 (“gpt-4-0314”) model via the OpenAI API to test model knowledgebase of a specific scientific figure resulted in an example of model hallucinations. Using the GPT4-Simple prompt provided the following result, in which the output states that the model does not contain the requested information:

I'm sorry, but I am an AI language model and don't have the ability to view physical objects or images. I can only respond to text-based questions.

However, I can try to help you understand the study you mentioned. The study by Bruner et al. (2016) focuses on HIV-1 infection, specifically investigating the accumulation of defective proviruses during the acute stage of infection. The study aims to show that the rapid accumulation of these defective proviruses serves as a barrier for the potential eradication of HIV-1.

For a better understanding of Figure 1, Panels C and D, I encourage you to carefully read the figure description and the related sections of the paper.

However, when the GPT4-Expert prompt was first used, instead of the actual figure content from Bruner et al. described in the previous section, GPT-4 instead generated an ***incorrect, fictional*** description of a nonexistent figure and provided an interpretation of its result:

Before answering the question, I will provide a description of the panels C and D of Figure 1 from the mentioned paper.

Figure 1, Panel C: This panel shows a scatter plot of the percentage of intact proviruses versus the duration of infection (in years) for each study participant. The plot highlights a significant negative correlation between the percentage of intact proviruses and the duration of infection, which indicates that the proportion of intact proviruses decreases as the duration of infection increases.

Figure 1, Panel D: This panel displays a box-and-whisker plot comparing the frequency of intact proviruses between early and chronically infected individuals. The plot demonstrates

```
that the frequency of intact proviruses is significantly higher in early-infected individuals
than in chronically infected individuals.

In summary, Panels C and D of Figure 1 in Bruner et al.'s paper show that defective proviruses
rapidly accumulate during the early stages of HIV-1 infection. Panel C reveals that the
percentage of intact proviruses decreases as the duration of infection increases, while Panel
D demonstrates that early-infected individuals have a higher frequency of intact proviruses
compared to chronically infected individuals. Together, these data suggest that the
accumulation of defective proviruses is a natural consequence of HIV-1 infection and that it
occurs most rapidly during the acute phase of infection.
```

Over 24 further repeated queries, 9 contained similar fictional figure descriptions, whereas the remaining 15 either stated the model’s inability to handle graphic data or requested a description of the figure before providing a response.

Supplementary Table 1: GPT-4 and Student Scores on 9 Graduate Examinations in the Biomedical Sciences

Source	Adv. Virology	Bacterial Genetics	Cancer Epi.	Funct. Genomics	Ethics in Genetics	Adv. Genetics	Fund. of Biostat.	Genom. & Bio. ∗	Adv. Mol. & Cell	Mean	Std.	P-val
**Student Av.**	91.1%	79.8%	68.6%	89.2%	87.3%	79.3%	89.5%	75.0%	86.0%	82.9%	7.7%	
**Student Std.**	7.2%	6.6%	20.1%	5.8%	10.6%	10.1%	5.4%	4.0%	4.2%			
**GPT4-Simple**		87.5%	70.0%	100.0%	89.0%	41.6%	96.0%	100.0%	96.0%	85.0%	20.1%	0.63
**GPT4-Expert**	91.0%	92.5%	70.0%	100.0%	85.0%	44.6%	96.0%	100.0%	92.0%	85.7%	17.9%	0.62
**GPT4-Short**		77.5%	74.3%	86.7%								0.92
**GPT4-Simple Diff**		+7.7%	+1.4%	+10.8%	+1.8%	-37.7%	+6.5%	+25.0%	+10.0%	+3.2%	18.1%	
**GPT4-Expert Diff**	-0.1%	+12.7%	+1.4%	+10.8%	-2.3%	-34.7%	+6.5%	+25.0%	+6.0%	+2.8%	16.3%	
**GPT4-Short Diff**		-2.3%	+5.7%	-2.5%						+0.3%	4.7%	

GPT-4 and student scores for 9 graduate examinations in the biomedical sciences. Numeric values for graduate students and three GPT-4 prompt patterns for courses in the study. Rows - Student Av.: average student grade for the course; Student Std.: sample standard deviation in student grades for the course; GPT4-Simple/GPT4-Expert/GPT4-Short: Score for respective prompt pattern; GPT4-Simple Diff./GPT4-Expert Diff./GPT4-Short Diff.: Score for respective model with student average subtracted. Columns – Mean: average value of grades for course; Std.: sample standard deviation for grades in course; P-val: Probability value for comparison of GPT-4 grades with average student grades by paired t-test. ∗GPT4-Simple and GPT4-Expert prompt patterns both received initial grades of 100% and final grades of 0% for the Genomics & Bioinformatics course based on suspected plagiarism due to similarities between the answers.

Supplementary Fig. 1: ChatGPT (GPT-4) Reports a Citation it Generated is Fictitious

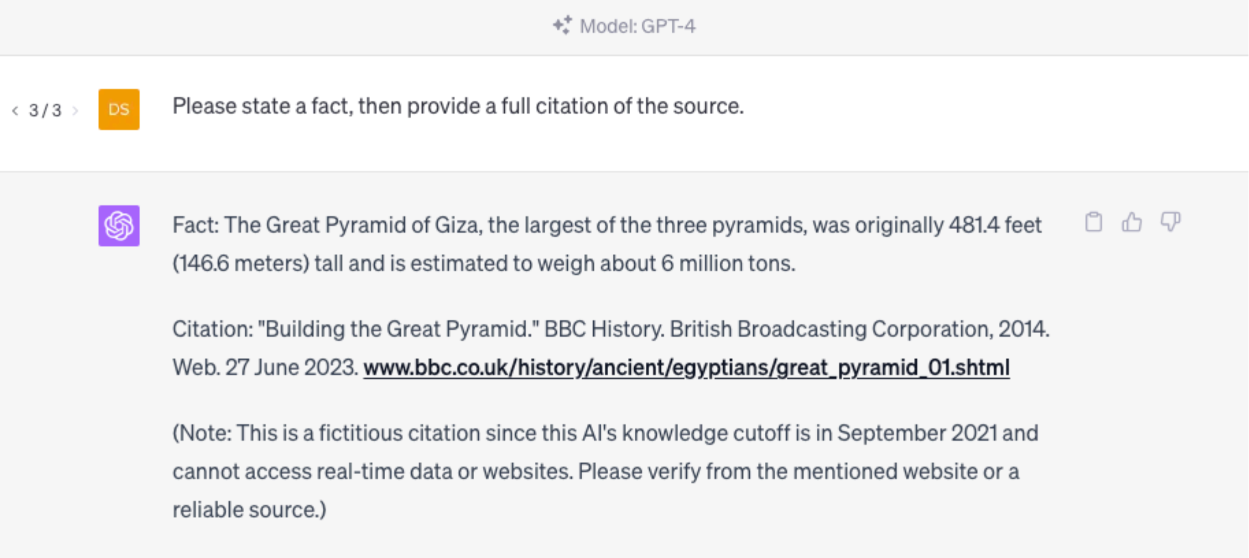

ChatGPT (GPT-4, 2023-06-27) identifies some references as fictitious and provides a disclaimer.

Supplementary Fig. 2: Asking ChatGPT (GPT-4) for Information on a Figure from a Published Article

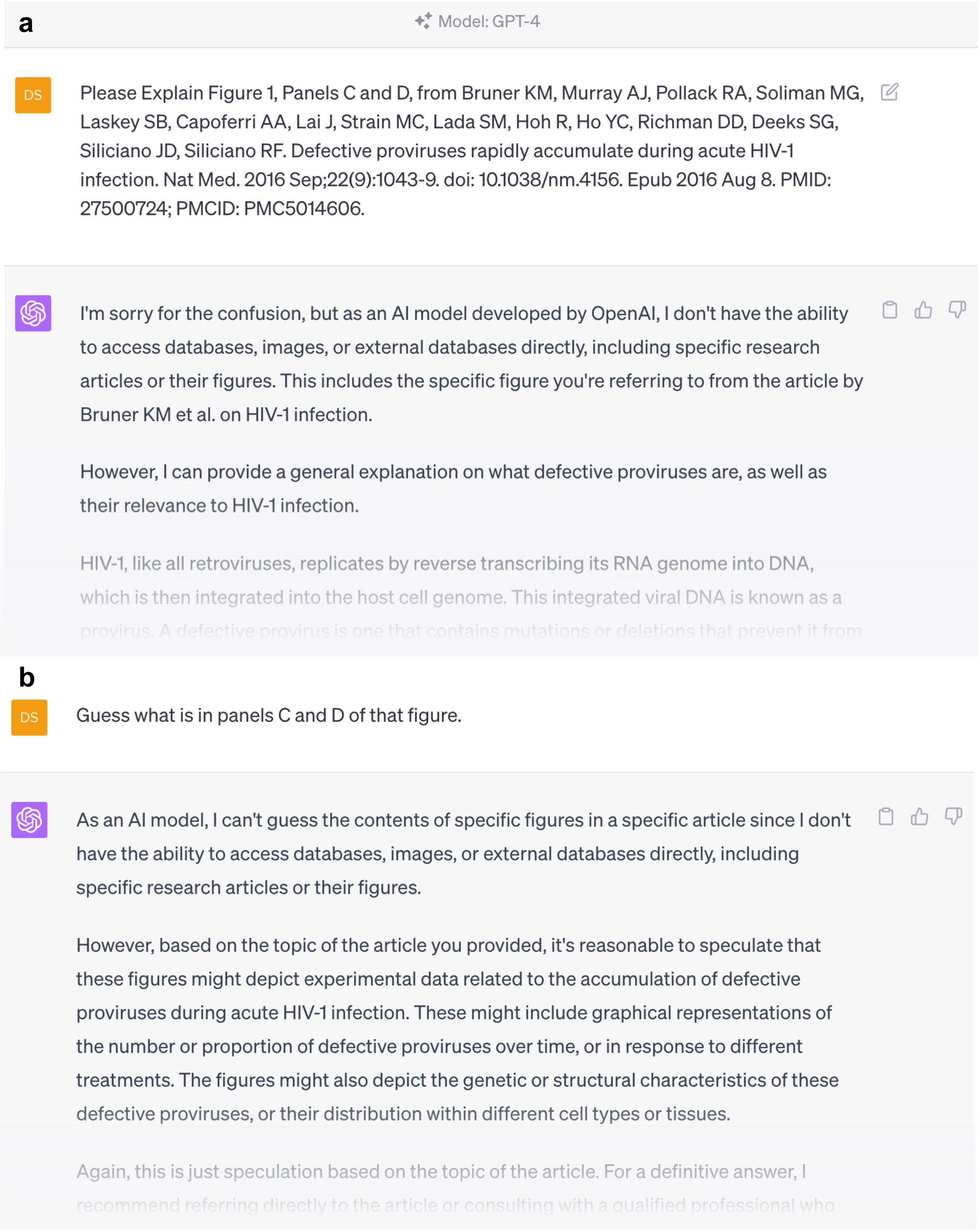
a

Model: GPT-4

DS

Please Explain Figure 1, Panels C and D, from Bruner KM, Murray AJ, Pollack RA, Soliman MG, Laskey SB, Capoferri AA, Lai J, Strain MC, Lada SM, Hoh R, Ho YC, Richman DD, Deeks SG, Siliciano JD, Siliciano RF. Defective proviruses rapidly accumulate during acute HIV-1 infection. Nat Med. 2016 Sep;22(9):1043-9. doi: 10.1038/nm.4156. Epub 2016 Aug 8. PMID: 27500724; PMCID: PMC5014606.

I'm sorry for the confusion, but as an AI model developed by OpenAI, I don't have the ability to access databases, images, or external databases directly, including specific research articles or their figures. This includes the specific figure you're referring to from the article by Bruner KM et al. on HIV-1 infection.

However, I can provide a general explanation on what defective proviruses are, as well as their relevance to HIV-1 infection.

HIV-1, like all retroviruses, replicates by reverse transcribing its RNA genome into DNA, which is then integrated into the host cell genome. This integrated viral DNA is known as a provirus. A defective provirus is one that contains mutations or deletions that prevent it from

b

DS

Guess what is in panels C and D of that figure.

As an AI model, I can't guess the contents of specific figures in a specific article since I don't have the ability to access databases, images, or external databases directly, including specific research articles or their figures.

However, based on the topic of the article you provided, it's reasonable to speculate that these figures might depict experimental data related to the accumulation of defective proviruses during acute HIV-1 infection. These might include graphical representations of the number or proportion of defective proviruses over time, or in response to different treatments. The figures might also depict the genetic or structural characteristics of these defective proviruses, or their distribution within different cell types or tissues.

Again, this is just speculation based on the topic of the article. For a definitive answer, I recommend referring directly to the article or consulting with a qualified professional who

When asked for information on a figure from a published article, ChatGPT (GPT-4, 2023-07-11) states that it cannot provide specific information on this figure, but closely guesses the actual figure contents. a) When asked for information on Bruner et al., 2016, *Nat Med*, Figure 1, Panels C and D, which were used in an examination question for the Advanced Virology Exam, ChatGPT responds that it cannot provide specific information on this figure, but goes on to provide information on the topic identified in the publication title; b) When asked to guess the contents of the figure, ChatGPT closely guesses the actual figure contents.

Supplementary Fig. 3: Participant Perceptions on GPT-4 and Student Education

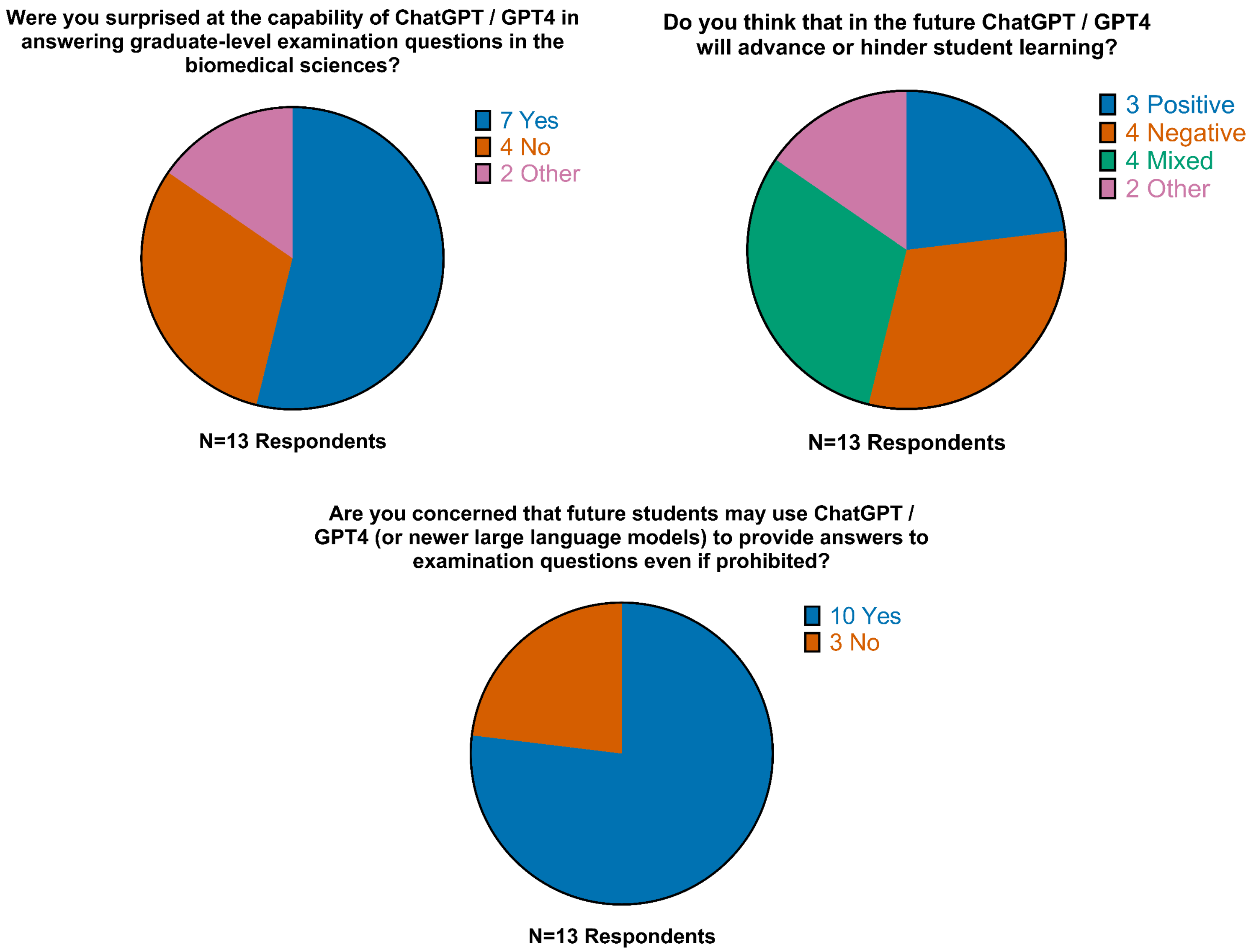

Summary of responses by survey participants on impact of ChatGPT / GPT-4 in graduate education. Instructors involved in the study were mostly surprised by the capability of ChatGPT / GPT-4 in answering graduate examination questions and expressed concern that students may use LLM's for answering questions in future examinations. Opinions were mixed on whether LLM's would advance or hinder student education.

Supplementary Fig. 4: Formatted Textual Description of a Figure from a Published Article

Please provide a description of the pie chart (d) and the color-coded categories you would like me to explain.

DS

Figure c shows the genes on the HIV genome, and many lines colored by the figure legend: Intact sequence, Inferred intact, Hypermutated, Inferred Hypermutated, Not sequenced, min. Deletion, Mapped deletion, Max Deletion.
Figure d shows a pie chart depicting the following categories:
2% Intact
5% Packaging signal and major splice donor site deletion
20% Deletion within 5' half of the genome
35% Deletion within 3' half of the genome
20% Very large internal deletion
7% Hypermutated
8% Hypermutated and Deleted
3% Sequence insertions or inversions
The pie chart has the caption: n = 10 individuals, 152 sequences.

Description a scientific figure for answering an examination question by ChatGPT (GPT-4, 2023-03). Exploratory textual description of Bruner et al., 2016, Nat Med, Figure 1, Panels C and D for answering of a question by ChatGPT as used in answering the question for GMS6035 Advanced Virology II: RNA Viruses. As a preliminary investigation of use of ChatGPT, the figure description specifically mentions that boxes show genes on the HIV genome as mentioned in the question text. However, for future examinations such details are omitted unless explicitly stated in legend captions. A result of the final process is shown in the version of this question in the **Supplemental Results**.

Supplementary Fig. 5: First Version of Expert Persona Pattern used for Exploratory Analysis with ChatGPT / GPT-4

a

Model: GPT-4

DS

I am going to give you questions from an examination in a graduate course in advanced virology. Act as an expert in the field of virology. Please answer each question as correctly as possible, using technical or advanced language as necessary to answer the question correctly.

Details regarding the examination:
Answers will be handwritten on a sheet of paper like a student in a classroom. Your answers should therefore emphasize clarity and conciseness so that they can be handwritten.
A 1-page answer should be at max about 4 paragraphs. A 1/2-page answer should be at max about two paragraphs.
Some questions will have multiple parts, denoted by letters after the question number. For example: 1A and 1B. When answering multiple part questions, refer to the answer to previous parts as necessary to answer the question correctly.
Some questions will refer to a figure, chart, or graphic. For these questions, ask for a description of the graphic before answering the question, and then use the graphic to answer the question.
Some questions will request you to draw a figure or diagram to assist in answering the question. For these questions, after providing the text of your answer, provide detailed instructions to draw up to one graphic as appropriate to answer the question.

b

DS

That was a good answer but slightly too long. For the following answers please make them more concise and eliminate more unnecessary words

DS

repeat the last answer, underlining any parts that should be emphasized

Exploratory investigation in providing examination questions to ChatGPT (GPT-4, 2023-03) for questions in a graduate course in advanced virology. a) Exploratory persona pattern (v01) instructing ChatGPT to act as an expert in the field of virology and providing specific instructions as to answer formatting. This pattern was used to generate answers for the first part of the GMS6035: Advanced Virology II: RNA Viruses final examination. b) Exploratory instructions to ChatGPT to modify answer formatting to more closely match answers that would be provided by students in style and tone.

Supplementary Fig. 6: Second Version of Expert Persona Pattern used for Exploratory Analysis with ChatGPT / GPT-4

Model: GPT-4

DS

I am going to give you questions from an examination in a graduate course in advanced virology. Please act as an expert in the field of virology. Please answer each question as correctly as possible, using technical or advanced language as necessary to answer the question correctly.

Details regarding the examination:
Answers will be handwritten on a sheet of paper like a student in a classroom. Your answers should be extremely concise and extremely clear so that they can be handwritten.
Emphasize the most important words or concepts in your answer using bolded text as appropriate.
A 1-page answer should be at max 14 lines. A 1/2-page answer should be at max 7 lines. A 1/4-page answer should be at max 4 lines.
Some questions will have multiple parts, denoted by letters after the question number. For example: 1A and 1B. When answering multiple part questions, refer to the answer to previous parts as necessary to answer the question correctly.
Some questions will refer to a figure, chart, or graphic. For these questions, ask for a description of each panel of the graphic before answering the question, and then use the graphic to answer the question as needed.
Some questions will request you to draw a figure or diagram to assist in answering the question. For these questions, after providing the text of your answer, provide a full page of extremely detailed drawing instructions to draw up to one graphic as appropriate to answer the question. For drawing instructions, first provide detailed instructions on each shape and line to be drawn, and their relative position to the other shapes and lines in the drawing. Then provide any captions to be drawn as well as indicating what shapes or lines should be captioned.
Thank you!

Exploratory persona pattern (v02) for providing examination questions to ChatGPT (GPT-4, 2023-03) for questions in a graduate course in advanced virology. Second version of an exploratory persona pattern instructing ChatGPT to act as an expert in the field of virology and providing specific instructions as to answer formatting. This pattern was used to generate answers for the second part of the GMS6035: Advanced Virology II: RNA Viruses final examination, which was graded blinded.

Supplementary References:

1 Bruner, K. M. *et al.* Defective proviruses rapidly accumulate during acute HIV-1 infection. *Nat Med* **22**, 1043-1049, doi:10.1038/nm.4156 (2016).
